# Heterogeneous effects of nursing staff wages on turnover: evidence from U.S. nursing homes using an instrumental variable approach

**DOI:** 10.1093/geront/gnag030

**Published:** 2026-03-23

**Authors:** Rohit Pradhan, Akbar Ghiasi, Ganisher Davlyatov, Robert Weech-Maldonado

**Affiliations:** School of Health Administration, College of Health Professions, Texas State University, San Marcos, Texas, United States; Department of Healthcare Administration, H-E-B School of Business and Administration, University of the Incarnate Word, San Antonio, Texas, United States; Department of Health Administration and Policy, Hudson College of Public Health, University of Oklahoma Health Campus, Oklahoma City, Oklahoma, United States; Department of Health Services Administration, School of Health Professions, University of Alabama at Birmingham, Birmingham, Alabama, United States

**Keywords:** Nursing homes, Turnover, Nursing staff

## Abstract

**Background and Objectives:**

Turnover among nursing staff (registered nurses, licensed practical nurses, and certified nursing assistants [CNAs]) is a long-standing challenge in nursing homes with significant implications for quality of care. This study aimed to examine the relationship between nursing staff wages and turnover.

**Research Design and Methods:**

We used national data from 2021 to 2023, linking multiple datasets including the Payroll-Based Journal and Medicare Cost Reports (*n* = 37,254). Turnover was modeled separately for each nursing staff type as a fractional outcome bounded between 0 and 1. The primary predictor variable was facility-level average hourly wage for each staff type. To address potential endogeneity and reverse causality, we used a two-stage residual inclusion instrumental variables approach with county-level average wages (excluding the index facility) as the instrument. Models controlled for facility-level organizational and county-level market characteristics, with fixed effects for county and year and cluster-robust standard errors at the facility level.

**Results:**

Higher CNA wages were significantly associated with lower turnover (*β* = −0.35, 95% CI [−0.59, −0.11], *p *= .005), indicating a 0.35 percentage point reduction in turnover per $1 increase in hourly wage. Wages were not significantly associated with turnover among licensed nurses.

**Discussion and Implications:**

Findings suggest that wage increases may be most effective for CNAs, while retaining licensed nurses likely requires complementary organizational and nonmonetary strategies. While nursing homes should strive to offer competitive wages to their staff, targeted reimbursement reforms may be necessary to overcome financial constraints.

Registered nurses (RNs), licensed practical nurses (LPNs), and certified nursing assistants (CNAs) serve as the primary caregivers in nursing homes, directly influencing resident care and outcomes (Abt Associates, 2023; Harrington et al., 2020). RNs serve as clinical leaders and are responsible for resident assessment, care planning, and the supervision of other nursing staff (Abt Associates, 2023; Mukamel et al., 2024). LPNs work closely with RNs to provide essential resident care, while CNAs have the most direct contact with residents (Bonner et al., 2022). Nursing staff are the cornerstone of care delivery in nursing homes, essential to meeting the needs of a population that is often physically and cognitively challenged. This is especially true within the United States (U.S.) system of long-term care, where fragmented financing through a mix of government and private payers complicates workforce recruitment and retention in the absence of a universal coverage model.

However, nursing homes have long struggled with high nursing staff turnover. A recent study using data from the Payroll-Based Journal (PBJ) reported alarmingly high median turnover rates of 102.9% for RNs, 79.8% for LPNs, and 98.8% for CNAs (Gandhi et al., 2021). Although turnover rates reported in other recent studies vary (Pradhan et al., 2025a; Zheng et al., 2022)—largely due to inconsistent definitions of turnover—it is a significant challenge in nursing homes. Turnover in nursing homes has consistently outpaced that of other healthcare settings, and the COVID-19 pandemic has only further intensified staffing pressures (Hassmiller & Wakefield, 2022).

Nursing staff turnover can adversely affect nursing home performance. Turnover has been associated with a higher number of quality of care deficiencies and increased mortality among nursing home residents (Antwi & Bowblis, 2018; Loomer et al., 2022; Shen et al., 2022). A study by Zheng et al. (2022) linked nursing staff turnover with poorer quality outcomes such as increased emergency department visits and a reduced likelihood of achieving higher star ratings in the Care-Compare: Five-Star Quality Rating System (Five-Star QRS). Pradhan et al. (2024b) found that the association between administrator turnover and Five-Star quality rating was fully mediated by RN turnover, highlighting its central role in shaping nursing home performance. Furthermore, increased turnover can be financially burdensome as nursing homes may be forced to divert resources toward recruitment and training, reducing the capacity to invest in quality improvement initiatives (Ghiasi et al., 2026; Pradhan et al., 2024a; Sharma & Xu, 2022).

An important strategy for attracting and retaining qualified nursing staff is to provide competitive wages and benefits (Heiks & Sabine, 2022). Efficiency wage theory suggests that offering higher wages can reduce employee turnover by increasing the opportunity cost of leaving, thereby enhancing staff retention and stability (Katz, 1986; Kim, 1999). The evidence on the relationship between nursing staff wages and turnover in nursing homes is mixed: studies generally have found little or no association for RNs and LPNs, but higher wages may be associated with modestly lower turnover among CNAs/nurse aides (Brunt et al., 2026; Kash et al., 2006; Sharma & Xu, 2022; Temple et al., 2011). However, prior studies are limited by single-state samples, cross-sectional designs, and the lack of strategies to address potential endogeneity. Utilizing tenets from efficiency wage theory, the aim of this study is to address this gap in the literature by examining the association between nursing staff wages and turnover in U.S. nursing homes.

We recognize that unmeasured factors—such as leadership quality (Williams et al., 2024), organizational culture (Banaszak-Holl et al., 2015), or organizational factors (Gandhi et al., 2021)—may confound the observed relationship between nursing staff wages and turnover. Additionally, reverse causality is a significant methodological challenge, as facilities with higher turnover may offer lower wages due to financial strain or, conversely, raise wages in response to staffing instability. To address these concerns, this study uses an instrumental variable (IV) approach that isolates exogenous variation in nursing staff wages and strengthen causal inference. In addition, the study uses nationally representative, longitudinal data from 2021 to 2023, enhancing the generalizability of findings and allowing for stronger temporal inference.

Findings from this study could inform wage-setting decisions and help policymakers and administrators balance compensation strategies with financial sustainability. These insights are particularly timely given the growing financial pressures facing nursing homes (Brunt et al., 2024; Orewa et al., 2024) and the increased policy focus on staffing requirements to ensure high-quality care for residents.

## Conceptual model

The relationship between nursing staff wages and turnover in nursing homes can be understood through the lens of efficiency wage theory which posits that employers may pay above-market wages to improve staff retention, job satisfaction, and productivity (Akerlof, 1982, 1986). In the context of nursing homes, efficiency wage theory suggests that higher nursing staff wages enhances retention by raising the opportunity cost of leaving (Shapiro & Stiglitz, 1984).

Applying the efficiency wage framework, there are several potential mechanisms through which higher nursing staff wages may reduce turnover. First, higher wages may reduce voluntary turnover by increasing the financial penalty of quitting—employees are less likely to leave a job that pays above their next-best alternative (Shapiro & Stiglitz, 1984). This mechanism is particularly relevant given the existing gap between wages in nursing homes and hospitals (Bureau of Labor Statistics, 2024), which can incentivize movement toward higher-paying care settings (Gandhi et al., 2021). Higher wages may also enhance morale and loyalty, as better compensation signals that employees are valued, fostering stronger commitment to the organization. Finally, competitive wages may help nursing homes attract and retain more qualified applicants, who are less likely to exit once employed. Evidence from the long-term care sector supports these mechanisms: prior studies have found that higher wages are associated with improved retention among nursing staff in nursing homes (Kash et al., 2006; Sharma & Xu, 2022).

We recognize that wages are not the sole determinant of turnover, and other factors can confound the observed relationship between pay and retention. Facility characteristics such as ownership type and size may influence both wage-setting and retention; for example, for-profit and chain-affiliated nursing homes often report higher turnover than nonprofit or independent facilities (Castle & Engberg, 2006). Staffing levels are also important, as inadequate nurse staffing increases workloads and stress for remaining staff, which can contribute to higher turnover in nursing homes (Temple et al., 2011). Along similar lines, market conditions such as competition among nursing homes can affect turnover. In counties with greater nursing home competition, facilities may face stronger pressure to retain staff, as competing providers can draw away nurses with more attractive wage offers or working conditions. By incorporating these potential confounders, our model aims to isolate the direct effect of nursing staff wages on turnover. The expectation, consistent with efficiency wage theory, is that higher wages will be associated with lower turnover even after accounting for these contextual factors.

## Method

### Data sources

Our study utilized the following secondary datasets for the period 2021–2023: Payroll-Based Journal (PBJ), Medicare Cost Reports, Provider of Service (POS) File, Area Health Resources File (AHRF), and Post-Acute Care and Hospice Provider Utilization and Payment Public Use Files (PAC PUF). The PBJ provides detailed auditable nursing home staffing data and was used to calculate the annual nursing staff turnover rates. The Medicare Cost Reports were used to calculate the average nursing staff wages. The POS and AHRF were used to derive organizational and market-level variables. The PAC PUF was used to calculate the Hierarchical Condition Category (HCC) risk score. Datasets were merged using the Centers for Medicare & Medicaid Services (CMS) Certification Number (CCN) for facility-level files and the Federal Information Processing Standard (FIPS) code for county-level merges. The final analytic data file included 37,254 facility-year observations, from 12,874 unique NHs. All independent variables, including facility-level wages and covariates, were lagged by one year relative to turnover to mitigate simultaneity bias. Thus, predictors measured for 2021–2023 were used to explain turnover during 2022–2024.

### Variables

Response variable: The primary outcome variable was nursing staff turnover rate, calculated separately for RNs, LPNs, and CNAs. We used the PBJ daily hours file to compute facility-level annual turnover for the specified nursing group (RNs: job codes 5–7; LPN/LVN: 8–9; CNA/aid categories: 10–12). Employment “spells” were constructed for each facility–employee: a new spell began on the first observed workday and any time a gap of at least 90 consecutive calendar days with no recorded hours occurs.

Eligibility for inclusion in the denominator required the spell’s first workday to fall within the baseline quarter or the first two quarters of the measurement year and at least 120 total hours worked within the first 90 calendar days after the start day. The numerator counted eligible spells with a separation, defined as the start of a ≥90-day no-work gap that begins within the measurement year. Annual turnover for a facility is the number of separations among eligible spells divided by the number of eligible spells, multiplied by 100. To mirror CMS publication practice, facility-year estimates with fewer than five eligible nurse spells were suppressed and excluded from pooled summaries.

Predictor variable: The independent variables were average hourly wages for RNs, LPNs, and CNAs, calculated separately at the facility level and expressed in dollars per hour. The Medicare Cost Reports provide the total employee compensation costs for directly employed nursing staff, including both wages and benefits. For each staff type, we calculated the average hourly cost by dividing total compensation costs by the total number of hours worked (Bowblis et al., 2024). The wages were adjusted for inflation using the Consumer Price Index to ensure comparability over time (Feng et al., 2010).

Confounding variables: We controlled for facility-level and community-level characteristics of nursing homes that may affect turnover (Pradhan et al., 2024b; Sharma & Xu, 2022). Facility-level control variables included the following: nursing staff (RN, LPN, and CNA) hours per resident day (PRD), size (resident count), and ownership. The ownership variable was specified as a categorical measure reflecting four combinations of ownership and chain affiliation: for-profit chain (FP chain), FP independent, not-for-profit chain (NFP chain), and NFP independent (Weech-Maldonado et al., 2012). We also controlled for facility-level occupancy rate (percentage of occupied beds) and payer mix (percentages of Medicare, Medicaid, and private-pay residents). To account for resident complexity, we used the HCC risk score, a CMS-developed risk adjustment index based on residents’ documented diagnoses (AAFP, 2023).

Community-level control variables were calculated at the county level: uninsurance rate (percentage of individuals under age 65 without health insurance), percentage of the population 65 years and older, poverty rate (percentage of individuals living below the federal poverty level), household income (median household income at the county level), Medicare Advantage (MA) penetration (percentage of Medicare beneficiaries in MA), and market competition. Competition refers to the degree to which nursing homes in a market compete for residents and resources. It was assessed using the Herfindahl–Hirschman Index (HHI), a metric that evaluates market concentration within an industry. HHI was calculated by summing the squares of each nursing home’s market share based on inpatient days, with values ranging between 0 and 1 (Feng et al., 2006). Higher values indicate more concentrated markets (less competition), while lower values reflect less concentrated markets (higher competition).

### Analysis

The analysis was conducted at the facility level. Descriptive statistics were used to summarize the dependent, independent, and control variables, including means and standard deviations for continuous variables and frequencies and percentages for categorical variables. We checked for potential multicollinearity among control variables using variance inflation factors (VIF) from the regression models. The mean VIF was 1.71 (range 1.06–3.45), well below commonly used thresholds, indicating no evidence of problematic multicollinearity among the variables.

Endogeneity, particularly reverse causality, is a key concern when examining the relationship between nursing staff wages and turnover. Facilities with high turnover may raise wages to attract staff, while those with more stable staffing models may offer lower wages. In such cases, the observed relationship between wages and turnover may reflect a facility’s reaction to staffing needs rather than the causal effect of wages on turnover. In addition, unobserved factors such as leadership quality or workplace culture may simultaneously affect both compensation policies and turnover, introducing omitted variable bias. These forms of endogeneity can bias estimates and obscure the true relationship between wages and turnover.

To address this concern, we employed an IV approach to obtain a more accurate estimate of the causal effect of wages on turnover. Adapting the approach of Mukamel et al. (2024), the IV used in this study was the average county-level wage for the same staff category (RNs, LPNs, or CNAs), excluding the index facility. Nursing homes within the same county operate in a shared labor market shaped by local economic conditions, labor supply, and regional competition. As a result, county-level average nursing staff wages are likely to be correlated with the wage levels set by individual facilities, satisfying the relevance condition for instrument validity.

Importantly, average nursing staff wages at other facilities in the same county should not directly affect turnover at the index facility, except through their influence on that facility’s own wage setting. This satisfies the exclusion restriction, as the instrument should influence turnover only indirectly through its effect on facility-level wages. While we acknowledge the possibility that regional wages may reflect broader labor market shocks—including pandemic-related stressors, we mitigate this concern by including county-level fixed effects in all models. By isolating exogenous variation in wages driven by external labor market conditions, the IV approach helps address both reverse causation and unmeasured confounding.

We modeled turnover as a proportion bounded between 0 and 1, using the facility-level turnover rate. All predictors were lagged by one year relative to turnover to mitigate simultaneity bias. We estimated the causal effect of wages on turnover with a two-stage residual inclusion (2SRI) control-function approach appropriate for a fractional outcome. In the first stage, we estimated a linear wage equation with county and year fixed effects using high-dimensional fixed effects regression. The dependent variable was inflation-adjusted hourly wage; the excluded instrument was the county-level average hourly wage computed excluding the index facility. The first-stage specification included all independent variables, with county and year fixed effects, and standard errors clustered at the facility level. We saved the first-stage residual to form the control function.

In the second stage, we fit a fractional logit model for the turnover proportion, including the observed wage, the first-stage residual, and the same covariates and fixed effects as above. The coefficient on the residual provides a direct test of remaining endogeneity; we used a Wald test of the null that the residual term equals zero. For interpretability, we reported average marginal effects of wages on turnover, expressing effects as percentage-point changes in turnover per $1 change in hourly wage while holding other regressors at their observed values.

To accommodate within-facility dependence over time, we computed cluster-robust standard errors at the facility level for both the first-stage wage equations and the second-stage fractional-logit models. All models include year fixed effects to capture common temporal shocks and county fixed effects to account for time-invariant community factors. All analyses were conducted in Stata version 19.5.

## Results

The descriptive statistics (Table 1) provide an overview of the key variables analyzed in this study. Mean (*SD*) turnover was high—RNs: 56.5% (29.3), LPNs: 58.4% (26.3), and CNAs: 62.8% (18.1). Inflation-adjusted hourly wages averaged $48.88 (11.16) for RNs, $38.27 (8.01) for LPNs, and $23.44 (5.48) for CNAs. Staffing intensity averaged 0.64 (0.37) RN HPRD, 0.90 (0.34) LPN HPRD, and 2.21 (0.53) CNA HPRD. Facilities had an average of 82 residents (*SD* = 46.8). Ownership was predominantly FP chain affiliated, while Medicaid was the largest payer source, comprising over half of the resident population. The average resident acuity (HCC) was moderate. Community-level characteristics indicated that nursing homes operated in markets with a 45% MA penetration rate, 18% of the population comprised of older adults, and 13% of the population below the federal poverty line. HHI indicated that markets were highly competitive (0.15).

**Table 1:** Descriptive statistics of the sample (*N* = 37,254 facility-year observations).

Panel A: scale variables	Mean (*SD*)
**Nursing staff turnover (proportion)**	
RN	56.51 (29.26)
LPN	58.43 (26.29)
CNA	62.83 (18.13)
**Nursing staff wages per hour ($/hour)**	
RN	48.88 (11.16)
LPN	38.27 (8.01)
CNA	23.44 (5.48)
**Nursing staff hours per resident day (hours)**	
RN	0.64 (0.37)
LPN	0.90 (0.34)
CNA	2.21 (0.53)
**Size (resident count)**	82.25 (46.81)
**Payer mix (%)**	
Medicare pay residents	13.55 (12.37)
Medicaid pay residents	55.01 (25.82)
Private pay residents	31.57 (22.66)
**HCC risk score**	2.66 (0.66)
**Uninsured rate (%)**	8.27 (4.34)
**County population 65+ (%)**	17.99 (4.08)
**Poverty rate (%)**	12.60 (4.49)
**Household income ($)**	74,322 (19,421)
**Medicare advantage penetration (%)**	44.79 (12.95)
**Competition (HHI; 0-1)**	0.15 (0.15)
**Panel B: categorical variables**	***n* (%)**
**Ownership**	
Not-for-profit independent	5,843 (15.70)
Not-for-profit chain	4,730 (12.70)
For-profit independent	11,572 (31.10)
For-profit chain	15,109 (40.50)

*Note*. Wages and household income in 2023 U.S. dollars. RN = registered nurse; LPN = licensed practical nurse; CAN = certified nursing assistant; HCC = hierarchical condition category; HHI = Herfindahl–Hirschman index.

Table 2 presents average marginal effects (AMEs) from the fractional-logit 2SRI models, expressed as percentage-points changes in turnover per $1 increase in hourly wage. RN wages were not associated with RN turnover, AME = +0.013 percentage-point per $1 (95% CI −0.145 to +0.171, *p* = .873). LPN wages were also not associated with LPN turnover, AME = −0.113 percentage-point per $1 (95% CI −0.347 to +0.122, *p* = .346). In contrast, higher CNA wages were linked to lower CNA turnover, AME = −0.350 percentage-point per $1 (95% CI −0.592 to −0.107, *p* = .005). The full first- and second-stage estimates from the two-stage residual inclusion models are presented in Supplementary Tables 1–3.

**Table 2:** Two-stage residual inclusion estimation (Stage 2): relationship between nursing staff wages and turnover (*N* = 37,254).

Variables	Registered nurse	Licensed practical nurse	Certified nursing assistant
	**Average marginal effects (*p*-value)**		
**Nursing staff wages per hour ($/hour)**	0.010 (.904)	−0.119 (.320)	−0.350 (.005)
**Nursing staff hours resident day (hours)**	−3.597 (.401)	−0.175 (.756)	−2.085 (<.001)
**Size (resident count)**	−0.035 (<.001)	−0.022 (<.001)	−0.008 (.005)
**Ownership**			
Not-for-profit independent	reference	reference	reference
Not-for-profit chain	0.891 (.171)	1.807 (.004)	0.814 (.048)
For-profit independent	2.473 (<.001)	1.959 (<.001)	1.957 (<.001)
For-profit chain	1.751 (.002)	0.986 (.056)	0.735 (.049)
**Payer mix (%)**			
Private pay residents	reference	reference	reference
Medicare pay residents	0.011 (.506)	−0.047 (.003)	−0.016 (.164)
Medicaid pay residents	0.034 (<.001)	0.014 (.061)	0.006 (.273)
**HCC risk score**	2.375 (<.001)	1.816 (<.001)	1.281 (<.001)
**Uninsured rate (%)**	0.252 (.633)	0.151 (.751)	0.215 (.452)
**County population 65+ (%)**	1.895 (<.001)	1.410 (.003)	1.471 (<.001)
**Poverty rate (%)**	0.083 (.740)	0.032 (.882)	0.141 (.290)
**Household income ($)**	0.001 (.123)	0.001 (.081)	0.001 (.862)
**Medicare Advantage penetration (%)**	0.437 (.008)	0.417 (.004)	0.346 (<.001)
**Competition (HHI)**	−3.559 (.144)	−1.242 (.559)	0.437 (.723)

*Note*. Wages and household income in 2023 U.S. dollars. HCC = hierarchical condition category; HHI = Herfindahl–Hirschman index.

As a robustness check, we replicated the turnover calculation approach used by Gandhi et al. (2021). The results were broadly consistent with our main findings, showing similar direction and magnitude of association between wages and turnover across staff types.

The ordinary least squares (OLS) association for RNs (−0.049; *p* = .03) attenuates to null under 2SRI (0.010; *p* = .904; Table 3). For LPNs, both approaches are null (OLS 0.004; *p* = .865; 2SRI −0.119; *p* = .320). In contrast, for CNAs the wage–turnover relationship is negative in both models and larger in magnitude under 2SRI (OLS −0.205; *p* < .001 vs 2SRI −0.350; *p* = .005), consistent with OLS attenuation from simultaneity or measurement error.

**Table 3:** Ordinary least squares regression of nursing staff turnover on wages.

Variables	Registered nurse	Licensed practical nurse	Certified nurse assistant
	***β*-coefficient (*p*-value)**		
**Nursing staff wages per hour ($/hour)**	−0.049 (.030)	0.004 (.865)	−0.205 (<.001)
**Nursing staff hours per resident day (hours)**	−4.059 (<.001)	−0.049 (.921)	−2.020 (<.001)
**Size (resident count)**	−0.037 (<.001)	−0.028 (<.001)	−0.014 (<.001)
**Ownership**			
Not-for-profit independent			
Not-for-profit chain	0.541 (.383)	1.486 (.009)	0.580 (.135)
For-profit independent	2.036 (<.001)	2.023 (<.001)	2.250 (<.001)
For-profit chain	1.630 (.002)	0.930 (.046)	1.012 (.002)
**Payer mix (%)**			
Private pay residents			
Medicare pay residents	0.027 (.094)	−0.040 (.006)	−0.009 (.347)
Medicaid pay residents	0.035 (<.001)	0.016 (.029)	0.011 (.022)
**HCC risk score**	2.264 (<.001)	1.630 (<.001)	1.084 (<.001)
**Uninsured rate (%)**	0.120 (.028)	0.166 (.002)	0.083 (.041)
**County population 65+ (%)**	−0.128 (.019)	−0.112 (.036)	−0.182 (<.001)
**Poverty rate (%)**	−0.330 (<.001)	−0.478 (<.001)	−0.426 (<.001)
**Household income ($)**	0.001 (.088)	0.001 (<.001)	0.001 (<.001)
**MA penetration (%)**	−0.037 (.023)	−0.061 (<.001)	−0.065 (<.001)
**Competition (HHI)**	−10.421 (<.001)	−7.006 (<.001)	−4.160 (<.001)

*Note*. Wages and household income in 2023 U.S. dollars. HCC = hierarchical condition category; HHI = Herfindahl–Hirschman index; MA = Medicare advantage.

Instrument validity and diagnostics indicated strong identification. In the first-stage equations with county and year fixed effects, CCN-clustered standard errors, and a comprehensive covariate set, the county-level average hourly wage (computed excluding the index facility) was a powerful predictor of facility wages: RN: −0.617 (SE 0.020), |*t*| = 30.10 (*F* ≈ 906); LPN: −0.568 (SE 0.017), |*t*| = .77 (*F* ≈ 1,140); CNA: −0.467 (SE 0.018), |*t*| = 26.38 (*F* ≈ 696).

In the second-stage fractional logit models with the control-function residual, tests of the residual term did not reject exogeneity for RNs [*χ*^2^(1) = 0.71, *p* = .401] or LPNs [χ^2^(1) = 0.78, *p* = .379] or CNAs [χ^2^(1) = 2.87, *p* = .090], suggesting no material residual endogeneity after adjustment for covariates and fixed effects.

Several covariates were significantly associated with nursing staff turnover after IV adjustment. Across nursing staff types, larger facilities had lower turnover (RN −0.035 per additional resident, LPN −0.022, CNA −0.008; all *p* < .01). Staffing intensity was staff type-specific: RN and LPN HPRD were not distinguishable from zero (*p* = .401 and 0.756), while higher CNA HPRD was associated with lower CNA turnover (−2.085 per 1 HPRD; *p* < .001). Ownership remained a strong predictor: relative to NFP independent homes, FP independent facilities had higher turnovers for all nursing staff types (RN +2.47, LPN +1.96, CNA +1.96; all *p* < .001), and FP chain facilities were also higher (RN +1.75, CNA +0.74; *p* ≤ .049). NFP chain status was higher for LPN (+1.81; *p* = .004) and CNA (+0.81; *p* = .048), but not for RN (*p* = .171). Payer mix showed that a greater Medicaid share corresponded to higher RN turnover (+0.034; *p* < .001); a higher Medicare share was linked to lower LPN turnover (−0.047; *p* = .003). Resident acuity (HCC) was positively associated with turnover for all staff types (RN +2.38, LPN +1.82, CNA +1.28; all *p* < .001).

County-level variables were broadly consistent across staff types: older population share was positively associated with turnover (RN +1.90, LPN +1.41, CNA +1.47; *p* ≤ .003). Medicare Advantage penetration was positively associated with turnover for all staff types (RN +0.44, LPN +0.42, CNA +0.35; *p* ≤ .008).

## Discussion

The primary objective of this study was to examine the relationship between nursing staff wages and turnover in U.S. nursing homes. Using an instrumental variable approach to address concerns about endogeneity and reverse causality, we isolated exogenous variation in wages and observed mixed associations. While higher wages were associated with lower turnover among CNAs, the associations for RNs and LPNs were not statistically significant after adjustment. These findings contributes to the growing body of research on nursing home workforce dynamics, particularly turnover (Brunt et al., 2026; Gandhi et al., 2021; Pradhan et al., 2024b; Sharma & Xu, 2022).

The mixed pattern of findings in this study is broadly consistent with both efficiency wage theory and prior empirical work in nursing homes. Efficiency wage theory posits that higher wages can reduce turnover by raising the opportunity cost of quitting and by signaling organizational investment in staff (Katz, 1986; Kim, 1999). Our results suggest that these mechanisms may operate most strongly for CNAs, who typically earn lower baseline wages, have fewer alternative benefits, and face more limited employment options than licensed nurses. Empirically, prior studies have often reported little or no association between wages and turnover for RNs and LPNs (Sharma & Xu, 2022; Temple et al., 2011), yet have found that higher wages are associated with lower turnover among CNAs (Brunt et al., 2026; Kash et al., 2006). Wage-turnover elasticity appears to be greater for CNAs than licensed nurses, underscoring the importance of staff-type-specific analyses rather than treating “nursing staff” as a homogeneous group.

The absence of a significant wage–turnover association for licensed nurses should also be interpreted in light of evidence that nonwage factors play an important role in turnover. Studies have shown that turnover is strongly influenced by organizational and environment factors such leadership style and local labor market characteristics (Castle & Engberg, 2006; Donoghue & Castle, 2007, 2009). Licensed nurses typically have higher educational attainment, greater professional autonomy, and more diverse employment opportunities than CNAs, which can that incremental wage differences may be less salient for their retention decisions relative to conditions such as moral distress, schedule control, and perceived respect in the workplace (Aloisio et al., 2021; Krein et al., 2022). In this context, our null findings for licensed nurses do not imply that wages are irrelevant, but rather that wage increases of the magnitude observed in our data may be insufficient on their own to change turnover behavior without concurrent improvements in work environment and organizational support.

Although the associations between CNA wages and turnover may appear modest at first glance, particularly when interpreted per-dollar increases, small improvements can have meaningful implications at the facility level. In this study, a one-dollar increase in CNA hourly wages was associated with a 0.35 percentage points reduction in turnover. Although the estimated effect is modest in absolute terms, even small wage increases could help reduce CNA turnover in an industry where turnover rates remain exceptionally high (Gandhi et al., 2021). Prior research indicates that lower nursing staff turnover is associated with improved quality of care in nursing homes (Gandhi et al., 2021). Importantly, nursing staff turnover is now a component of the staffing quality domain in the Five-Star QRS (Centers for Medicare & Medicaid Services, 2022), meaning that improvements in retention can have a direct and measurable impact on a facility’s star rating and public profile, and may help attract a more favorable payer mix and strengthen financial sustainability (Park et al., 2011).

Beyond wages, ownership structure emerged as a significant predictor of nursing staff turnover, with for-profit chain facilities exhibiting the highest turnover rates. These findings align with prior research suggesting that for-profit ownership, particularly within chains, may prioritize cost containment strategies that contribute to poorer nursing home outcomes (Harrington, 2013; Harrington et al., 2012). Concerns may be further amplified in the context of private equity ownership, which has been linked to reduced nursing staff levels and lower quality of care in nursing homes (Gupta et al., 2024). The organizational context in which nursing homes operate is important, as structural characteristics can significantly influence workforce outcomes.

## Practice implications

This study highlights the importance of considering staff-type differences when designing strategies to reduce nursing staff turnover in nursing homes. Our findings suggest that wages may be more salient for CNAs compared to licensed nurses. For nursing home leaders, this implies that competitive wage structures for CNAs should be an important component of workforce strategies, especially in markets were CNA wages lag behind other service sector employers. Recent national evidence suggests that, in addition to higher wages, increased spending on fringe benefits (e.g., health insurance, childcare) may be an especially cost-effective strategy for reducing nurse aide turnover (Brunt et al., 2026). Although financial may constrain wage increases (Brunt et al., 2024), stabilizing the nursing workforce is essential for operational continuity and maintaining high-quality care (Gandhi et al., 2021). This is particularly important in light of the upcoming expansion of the Skilled Nursing Facility Value-Based Purchasing Program, which increasingly ties reimbursement to quality performance outcomes (Centers for Medicare & Medicaid Services, 2024). Moreover, reducing turnover could lessen recurring costs related to constant recruitment, onboarding, and staff replacement (Sharma & Xu, 2022). It may also decrease reliance on agency or contract nurses, which can negatively impact both nursing home quality (Pradhan et al., 2025b) as well as financial performance (Lord et al., 2025).

At the same time, it may be important to emphasize nonmonetary improvements to enhance the retention of licensed nurses. Strengthening staffing adequacy, reducing workload pressures, improving schedule flexibility, and fostering supportive supervision may have a greater impact on licensed nurse retention than incremental wage increases. Programs that promote professional growth and shared decision-making can further reinforce commitment to the organization (Abt Associates, 2023).

This study reinforces the importance of labor market context in shaping facility-level outcomes. Our instrument—county-level average wages—reflects broader market conditions that facilities should respond to when recruiting and retaining staff. These findings suggest that nursing homes operating in more competitive or higher-cost labor markets may require tailored staffing and compensation strategies to maintain workforce stability.

Policy support may be necessary to incentivize nursing homes to prioritize reducing nursing staff turnover rates. For instance, the National Academies of Sciences, Engineering and Medicine (2022) has recommended designating a specific percentage of Medicare and Medicaid payments for direct-care services, including staffing levels, wages, and benefits, to ensure adequate investment in nursing home workforce development. Many states have implemented wage pass-through policies that earmark a portion of Medicaid payments specifically for direct care worker compensation (RTI International, 2024). Such initiatives could be expanded—and potentially coordinated at the federal level—to ensure more consistent support for CNA wages and benefits across states. Targeted incentives may also be warranted for facilities in rural or underserved areas, which face persistent labor market challenges (Abt Associates, 2023).

The new CMS minimum nursing staff regulations are caught in legal purgatory (Chidambaram, 2025) and face significant practical barriers, including the ongoing acute shortage of nursing staff in the U.S. healthcare system (Dobbs et al., 2025; Haddad et al., 2018). In this context, strengthening nursing staff retention may offer a more practical and sustainable approach to achieving the same goals of improved continuity of care and quality. Supporting the existing workforce through competitive wages and better working conditions could help nursing homes meet policy expectations without relying solely on regulatory mandates.

The turnover rates reported in this study are somewhat lower than rates reported in recent studies (Gandhi et al., 2021). This difference likely reflects inconsistencies in how turnover is defined and measured across studies. To improve transparency and support quality improvement, CMS should consider reporting separate turnover rates for LPNs and CNAs in the Five-Star QRS.

### Limitations and future research

This study has several limitations. First, while our IV strategy reduces concerns about reverse causality, the validity of our estimates depends on the assumption that the chosen instrument (county-level wages excluding the index facility) is not directly related to turnover for reasons other than through its effect on facility wages. While this is a common and defensible approach, it cannot be directly tested, and future research should explore alternative instruments to strengthen causal inference. Second, the administrative datasets used in this study do not capture key nonwage determinants of turnover, such as work environment, leadership quality, or burnout. Qualitative or mixed-methods research could help illustrate the mechanisms through which compensation influences turnover and identify complementary strategies beyond wages. Third, when merging wage and turnover data, there is potential for temporal misalignment, as these variables may be constructed over different reporting periods. This is a common challenge in nursing home research when using administrative datasets and should be considered when interpreting results. Future work should examine whether the effect of wage increases on RN turnover varies across facility characteristics (e.g., ownership, rurality) and workforce demographics (e.g., tenure, age). More research is also required to evaluate the effectiveness of Medicaid wage pass-through laws in improving the recruitment and retention of nursing staff in nursing homes.

## Conclusions

This study provides new evidence on the relationship between nursing staff wages and turnover in U.S. nursing homes. By using an IV approach with nationally representative data, we address concerns about reverse causality and unobserved confounding, offering more robust estimates of this relationship. The mixed findings of this study suggest that wage increases may be most effective for nurse aides, whereas retaining licensed nurses likely requires broader organizational and nonmonetary strategies. However, the financial constraints faced by many nursing homes, particularly those heavily reliant on Medicaid reimbursement, present significant challenges to implementing wage increases or even resource-intensive nonmonetary incentives. Structural and reimbursement reforms that support both fair compensation and improved working conditions are essential to ensuring the delivery of high-quality care and supporting the sustainability of the nursing home workforce.

## Supplementary Material

gnag030_Supplementary_Data

## Data Availability

Data, analytic methods, or materials utilized in this study are available to other researchers for replication purposes based upon reasonable requests. The study is not preregistered. Study data are publicly available. Payroll-Based Journal dataset: https://data.cms.gov/quality-of-care/payroll-based-journal-daily-nurse-staffing; Medicare Cost Reports: https://www.cms.gov/data-research/statistics-trends-and-reports/cost-reports; Provider of Service File: https://data.cms.gov/provider-data/dataset/4pq5-n9py?; Area Health Resources Files: https://data.hrsa.gov/topics/health-workforce/ahrf; Post-Acute Care and Hospice Provider Utilization and Payment Public Use Files: https://data.cms.gov/resources/medicare-post-acute-care-hospice-by-provider-and-service-methodology.
