# Supplementary material for: Heterogeneous effects of nursing staff wages on turnover: evidence from U.S. nursing homes using an instrumental variable approach
Source: Gerontologist. 2026 Mar 23;66(5):gnag030. doi: 10.1093/geront/gnag030 (PMC13131959; doi:10.1093/geront/gnag030)
Supplement: gnag030_Supplementary_Data [file gnag030_supplementary_data.pdf]

Heterogeneous Effects of Nursing Staff Wages on Turnover: Evidence from U.S. Nursing Homes Using an Instrumental Variable Approach

Rohit Pradhan*, PhD
Associate Professor
School of Health Administration
College of Health Professions
Texas State University
San Marcos, TX. USA
https://orcid.org/0009-0006-3762-7212

Akbar Ghiasi, PhD
Assistant Professor
Department of Healthcare Administration
H-E-B School of Business and Administration
University of the Incarnate Word
San Antonio, TX. USA
https://orcid.org/0000-0002-1477-4985

Ganisher Davlyatov, PhD
Assistant Professor
Department of Health Administration and Policy
Hudson College of Public Health
University of Oklahoma Health Campus
Oklahoma City, OK. USA
https://orcid.org/0000-0001-9410-9696

Robert Weech-Maldonado, PhD
Professor Emeritus
Department of Health Services Administration
School of Health Professions
University of Alabama at Birmingham
Birmingham, AL. USA
https://orcid.org/0000-0002-5005-0909

Supplementary Table 1: Two-stage residual inclusion estimation (Stage 1): Relationship between nursing staff wages and turnover (N =37,254)

Variables	Registered Nurse	Licensed Practical Nurse	Certified Nurse Assistant
	β-coefficient (p-value)		
Nursing staff hourly wage ($)	-0.617 (<0.001)	-0.568 (<0.001)	-0.467 (<0.001)
Nursing staff hours PRD (hours)	0.974 (<0.001)	0.578 (<0.001)	0.510 (<0.001)
Size (resident count)	0.011 (<0.001)	0.011 (<0.001)	0.007 (<0.001)
Ownership			
Not-for-profit independent	reference	reference	reference
Not-for-profit chain	0.266 (0.301)	0.127 (0.465)	0.249 (0.034)
For-profit independent	-1.988 (<0.001)	-1.153 (<0.001)	-1.111 (<0.001)
For-profit chain	-1.063 (<0.001)	-0.549 (<0.001)	-0.632 (<0.001)
Payer mix (%)			
Private pay residents	reference	reference	reference
Medicare pay residents	-0.031 (<0.001)	-0.013 (0.009)	-0.019 (<0.001)
Medicaid pay residents	-0.012 (<0.001)	-0.007 (0.002)	-0.009 (<0.001)
HCC risk score	0.190 (0.081)	0.216 (0.004)	-0.067 (0.183)
Uninsured rate (%)	-0.071 (0.540)	0.006 (0.938)	-0.044 (0.410)
County population 65+ (%)	0.916 (<0.001)	0.612 (<0.001)	0.384 (<0.001)
Poverty rate (%)	0.059 (0.340)	0.013 (0.745)	-0.029 (0.294)
Household income ($)	0.001 (0.09)	0.001 (0.210)	0.001 (0.012)
Medicare Advantage penetration (%)	-0.022 (0.599)	0.099 (<0.001)	0.079 (<0.001)
Competition (HHI)	1.608 (0.01)	1.877 (0.614)	0.162 (0.944)

Note: PRD: per resident day; HCC: Hierarchical Condition Category; HHI: Herfindahl-Hirschman Index
Wages and household income in 2023 US dollars.

Supplementary Table 2: Two-stage residual inclusion estimation (Stage 2): Relationship between nursing staff wages and turnover (N =37,254)

**Variables**	**Registered Nurse**	**Licensed Practical Nurse**	**Certified Nurse Assistant**
	Average marginal effects (95% confidence intervals)		
Nurse wages per hour ($/hour)	0.010 (-0.149 to 0.168)	-0.119 (-0.353 to 0.115)	-0.350** (-0.592 to -0.108)
Nurse hours per resident day (hours)	-3.597*** (-4.887 to -2.308)	-0.175 (-0.283 to 0.932)	-2.085*** (-2.626 to -1.544)
Size (resident count)	-0.035*** (-0.043 to -0.027)	-0.022*** (-0.030 to -0.014)	-0.008** (-0.014 to -0.002)
Ownership			
Not-for-profit independent	reference	reference	reference
Not-for-profit chain	0.891 (-0.385 to 2.166)	1.807** (0.580 to 3.035)	0.814* (0.008 to 1.621)
For-profit independent	2.473*** (1.316 to 3.630)	1.959*** (0.913 to 3.005)	1.957*** (1/188 to 2.726)
For-profit chain	1.751** (0.650 to 2.851)	0.986 (-0.025 to 1.996)	0.735* (0.008 to 1.462)
Payer mix (%)			
Private pay residents	reference	reference	reference
Medicare pay residents	0.011 (-0.022 to 0.045)	-0.047** (-0.078 to -0.015)	-0.016 (-0.039 to 0.007)
Medicaid pay residents	0.034*** (0.017 to 0.050)	0.014 (-0.001 to 0.029)	0.006 (-0.005 to 0.017)
HCC risk score	2.375*** (1.826 to 2.924)	1.816*** (1.291 to 2.341)	1.281*** (0.917 to 1.646)
Uninsured rate (%)	0.252 (-0.781 to 1.284)	0.151 (-0.777 to 1.078)	0.215 (-0.345 to 0.775)
County population 65+ (%)	1.895*** (0.842 to 2.948)	1.410** (0.465 to 2.355)	1.471*** (0.881 to 2.061)
Poverty rate (%)	0.083 (-0.404 to 0.569)	0.032 (-0.389 to 0.453)	0.141 (-0.120 to 0.403)
Household income ($)	0.001 (0.001 to 0.001)	0.001 (0.001 to 0.001)	0.001 (0.001 to 0.001)
Medicare Advantage penetration (%)	0.437** (0.116 to 0.757)	0.417** (0.130 to 0.704)	0.346*** (0.173 to 0.519)
Competition (HHI)	-3.559 (-8.338 to 1.218)	-1.242 (-5.409 to 2.925)	0.437 (-1.975 to 2.848)

Note: HCC=Hierarchical Condition Category; HHI=Herfindahl-Hirschman Index
p-value: * <0.05, ** <0.01, *** < 0.001
Wages and household income in 2023 US dollars.

Supplementary Table 3: Two-stage residual inclusion estimation (Stage 1): Relationship between nursing staff wages and turnover (N =37,254)

**Variables**	**Registered Nurse**	**Licensed Practical Nurse**	**Certified Nurse Assistant**
	β-coefficient (95% confidence intervals)		
Nursing staff hourly wage ($)	-0.617*** (-0.657 to -0.577)	-0.568*** (-0.601 to -0.535)	-0.467*** (-0.501 to -0.432)
Nursing staff hours PRD (hours)	0.974*** (0.451 to 1.498)	0.578*** (0.259 to 0.898)	0.510*** (0.374 to 0.645)
Size (resident count)	0.011*** (0.007 to 0.015)	0.011*** (0.009 to 0.014)	0.007*** (0.005 to 0.009)
Ownership			
Not-for-profit independent	reference	reference	reference
Not-for-profit chain	0.266 (-0.239 to 0.772)	0.127 (-0.213 to 0.466)	0.249* (0.019 to 0.479)
For-profit independent	-1.988*** (-2.424 to -1.552)	-1.153*** (-1.448 to -0.859)	-1.111*** (-1.312 to -0.910)
For-profit chain	-1.063*** (-1.474 to -0.652)	-0.549*** (-0.833 to -0.265)	-0.632*** (-0.827 to -0.436)
Payer mix (%)			
Private pay residents	reference	reference	reference
Medicare pay residents	-0.031*** (-0.045 to -0.017)	-0.013** (-0.023 to -0.003)	-0.019*** (-0.026 to -0.013)
Medicaid pay residents	-0.012*** (-0.019 to -0.006)	-0.007** (-0.011 to -0.002)	-0.009*** (-0.012 to -0.007)
HCC risk score	0.190 (-0.023 to 0.403)	0.216** (0.069 to 0.364)	-0.067 (-0.165 to 0.032)
Uninsured rate (%)	-0.071 (-0.297 to 0.155)	0.006 (-0.148 to 0.160)	-0.044 (-0.149 to 0.061)
County population 65+ (%)	0.916*** (0.657 to 1.176)	0.612*** (0.406 to 0.819)	0.384*** (0.267 to 0.501)
Poverty rate (%)	0.059 (-0.062 to 0.181)	0.013 (-0.066 to 0.093)	-0.029 (-0.082 to 0.025)
Household income ($)	0.001 (0.001 to 0.001)	0.001 (0.001 to 0.001)	0.001* (0.001 to 0.001)
Medicare Advantage penetration (%)	-0.022 (-0.104 to 0.060)	0.099*** (0.045 to 0.153)	0.079*** (0.043 to 0.115)
Competition (HHI)	1.608* (-7.551 to 10.767)	1.877 (-5.417 to 9.171)	0.162 (-4.377 to 4.700)

Note: PRD: per resident day; HCC=Hierarchical Condition Category; HHI=Herfindahl-Hirschman Index
p-value: * <0.05, ** <0.01, *** < 0.001
Wages and household income in 2023 US dollars
